# Availability, prices and affordability of selected antibiotics and medicines against non-communicable diseases in western Cameroon and northeast DR Congo

**DOI:** 10.1371/journal.pone.0227515

**Published:** 2020-01-07

**Authors:** Simon Schäfermann, Richard Neci, Edward Ngah Ndze, Fidelis Nyaah, Valentin Basolanduma Pondo, Lutz Heide

**Affiliations:** 1 Pharmaceutical Institute, Eberhard Karls University Tuebingen, Tuebingen, Germany; 2 Le Dépôt Central Médico-Pharmaceutique de la 8e CEPAC (DCMP), Bukavu, Democratic Republic of Congo; 3 Central Pharmacy, Cameroon Baptist Convention (CBC), Mutengene, Cameroon; 4 Central Pharmacy, Presbyterian Church in Cameroon (PCC), Limbe, Cameroon; 5 Centrale d’Approvisionnnement et de Distribution des Medicaments Essentiels (CADIMEBU), Bunia, Democratic Republic of Congo; Purdue University, UNITED STATES

## Abstract

Access to safe, effective and affordable medicines of good quality is included into the Sustainable Development Goals of the United Nations. Furthermore, WHO has developed a Global Action Plan with the aim to raise access to essential medicines against non-communicable diseases (NCDs) to 80%, and to improve their affordability. In order to contribute to the monitoring of progress towards these goals, the present study investigated the availability and affordability of seven antibiotics and six medicines against non-communicable diseases in the northeast of the Democratic Republic of Congo and the west of the Republic of Cameroon. Data on availability and prices of these medicines were collected in 60 different sites (34 in the DR Congo, 26 in Cameroon), including government health facilities, church health facilities, private pharmacies and informal vendors, as part of a study on medicine quality. The data were analyzed using a standardized procedure developed by WHO and Health Action International (HAI). Average availability of the investigated antibiotics ranged from 62% to 98% in the different types of facilities in both countries, including the informal vendors. Average availability for medicines against NCDs in the different types of facilities showed a higher variation in both countries, ranging from 11% up to 87%. The average availability of medicines against NCDs in government health facilities was only 33% in Cameroon, and as low as 11% in the DR Congo. In contrast, availability of medicines against NCDs in church health facilities in Cameroon was 70%, not far from the 80% availability goal set by WHO. Medicine prices were clearly higher in Cameroon than in the DR Congo, with median price ratios to an international reference price of 5.69 and 2.17, respectively (p < 0.001). In relation to the daily minimum wages in both countries, treatment courses with five of the seven investigated antibiotics could be considered as affordable, while in each country only one out of the five investigated medicines against NCDs could be considered as affordable. Especially generic medicines provided by government and church health facilities showed reasonable affordability in most cases, while originator medicines offered by private pharmacies were clearly unaffordable to a major part of the population. Despite some encouraging findings on the availability of antibiotics in both countries, the availability and affordability of medicines against NCDs urgently requires further improvements.

## Introduction

According to the WHO millions of people each year die unnecessarily from a disease or condition even though effective medicines or vaccines exist for its treatment [1]. This is also attributable to poor availability of essential medicines. Achieving access to safe, effective, quality and affordable medicines and vaccines for all is therefore one component of the target No. 3.8 of the Sustainable Development Goals of the United Nations [2]. Despite considerable efforts, the availability of medicines is still poor in many countries [3, 4]. A survey covering data from 25 low and middle income countries reported a 40% overall median availability of essential medicines in the public sector in 2014. [5]. Especially for the treatment of non-communicable diseases (NCDs) the availability of essential medicines is low, as a recent analysis of 30 surveys published by Ewen et al. [6] and a review by Robertson et al. [7] indicate. In 2010, a study investigated the availability of five cardiovascular medicines in 36 countries, reporting a mean availability of 26.3% in the public sector [8]. There is still a long way to go to reach the goal of 80% availability for medicines against NCDs as set by the WHO for 2025 [9]. But even when essential medicines are available, not every patient in low- and middle-income countries can afford them. Unaffordability of medicines can lead to non-adherence of patients to their treatment and aggravate a country’s burden of disease [10]. An evaluation of data from 36 countries showed in 2009 that treatments for acute and chronic diseases were unaffordable in many countries [11]. The above mentioned analysis of cardiovascular medicines in 2010 showed that treatment courses for chronic disease are especially unaffordable when combination therapies are required [8]. The assessment by Ewen et al. [6] also underlined that only in few low—and—middle income countries essential medicines against NCDs are both available and affordable.

Few surveys so far have investigated the availability and affordability of medicines in Cameroon, and even fewer in the Democratic Republic of Congo (DR Congo). In 2005, Preux et al. [12] investigated the availability of antiepileptic medicines in a very small survey in the Mifi Province in Cameroon and reported that 32 out of 33 patients were treated with a modern antiepileptic medicine. But these were frequently out of stock in hospital pharmacies and patients needed to buy them from private pharmacies and even from informal (illegal) vendors. A negative aspect of availability was reported by Becker et al. [13]. Injectable antibiotics in southwest Cameroon were readily available from market stalls and traditional healers at a price lower than in the government clinics. An investigation by Jingi et al. [14] revealed that in the West Region of Cameroon in 2014 a one month combination treatment for coronary heart diseases was as expensive as 41 days wages. In 2011, O’Connell et al. [15] investigated the availability of anti-malarial medicines in six countries including the Democratic Republic of Congo, with the encouraging finding that 82% of the public health facilities in DR Congo had a quality assured artemisinin-based combination therapy available. In 2014, the Ministry of Health of the DR Congo published a “Service Availability and Readiness Assessment” (SARA), including data on the availability of 20 essential medicines [16]. Overall, the availability was only 20%, and was especially low for medicines against non-communicable diseases. Notably, the two provinces with the highest average availability (35% and 30% respectively) were North-Kivu and South-Kivu, which have also been investigated in the present study.

Cameroon and the DR Congo show different political and financial characteristics: Cameroon is a lower-middle income country with a gross national income per capita (GNI per capita) of 3.315 US–Dollar (USD) per year. 23.8% of the total population live below the income poverty line of 1.90 USD per day. Life expectancy at birth is 58.6 years [17]. Despite a stable political history, in recent years Cameroon faces turmoil caused by attacks of the Boko Haram in the North and separatist movements in the anglophone regions [17]. The DR Congo is a low-income country with a GNI per capita of only 976 USDper year, less than 30% of the GNI of Cameroon. 77.1% of the total population live below the income poverty line, a three times higher proportion than in Cameroon. However the life expectancy at birth (60.0 years) is reported to be similar to that of Cameroon [18]. Following a long series of political conflicts, the outbreak of Ebola starting in 2018 in the province North-Kivu and an ongoing armed conflict, the situation for the population in the northeast part of DR Congo, where the present study was conducted, is grave [19].

The present data on the availability and prices of thirteen selected essential medicines were collected as part of a study on medicine quality in the western regions of Cameroon and the northeastern regions of the DR Congo. While the laboratory investigation of the collected medicine samples is still ongoing, we here present the results on availability, prices and affordability of medicines in government and church health facilities, private pharmacies and informal (illegal) vendors in northeast DR Congo and west Cameroon.

## Methods

### Selection of medicines

The study protocol followed the guidelines on the conduct of surveys of the quality of medicines, published by the WHO in 2016 [20], and the MEDQUARG guidelines [21]. For the study in the DR Congo seven antibiotics and five medicines against non-communicable diseases (NCDs) were selected. All medicines selected were part of the respective essential medicines list (EML) of both countries and, according to the information by our local partners, frequently used by health care providers. Since this study was carried out as part of a study on medicine quality, using both GPHF Minilab analysis (www.gphf.org) and analysis according to the United States Pharmacopeia (USP), the selection of medicines was also based on the availability of both a GPHF Minilab monograph and a USP monograph. Therefore, the included medicines were solid oral dosage forms of amoxicillin, amoxicillin/clavulanic acid, sulfamethoxazole/trimethoprim, ciprofloxacin, phenoxymethylpenicillin (penicillin V), metronidazole, doxycycline, metformin, atenolol, hydrochlorothiazide, furosemide and salbutamol (albuterol). For the study in Cameroon, the same medicines except atenolol were selected, since according to the local partners in Cameroon atenolol was not frequently used by healthcare providers in the Republic of Cameroon. This is also confirmed by the study of Jingi et.al [14] who reported that out of 11 investigated health facilities in Cameroon, the only one which stocked atenolol was an exceptionally well-stocked private pharmacy shop. Upon request by the local partners in Cameroon, the antidiabetic medicine glibenclamide (glyburide) was collected instead of the antihypertensive medicine atenolol in that country. All of the thirteen medicines were listed in the essential medicines lists (EML) of the Republic of Cameroon and the Democratic Republic of Congo [22, 23]. The preferred strength of the collected medicines is shown in Table 1. If this strength was unavailable, tablets or capsules of an another strength were sampled.

**Table 1:** Medicines included into this study and calculation of medicine amount of the preferred strength used for one course of treatment.

	Model disease	Strength [mg/tbl. or cps.]	Dosage regimen [tbl. or cps./day]	Treatment duration	Number of tbl./cps. for one course of treatment
Amoxicillin / clavulanic acid tbl.[28]	Adult respiratory infection	500 / 125	3	7 days	21
Amoxicillin tbl./cps. [25]	Adult respiratory infection	500	3	7 days	21
Ciprofloxacin tbl. [25]	Adult respiratory infection	500	2	7 days	14
Metronidazole tbl. [28]	Anaerobic infections	250	6	7 days	42
Penicillin V tbl. [28]	Adult respiratory infection	250	8	7 days	56
Doxycycline tbl./cps. [28]	Malaria	100	2	10 days	20
Sulfamethoxazole and trimethoprime tbl. [25, 28]	Respiratory tract infections	400 / 80	4	7 days	28
Metformin tbl. [28]	Diabetes	500	3	30 days	90
Glibenclamide tbl. [25]	Diabetes	5	2	30 days	60
Atenolol tbl.[25]	Hypertension	50	1	30 days	30
Salbutamol tbl.[28]	Chronic asthma	2	3	30 days	90
Furosemide tbl. [28]	Oedema	40	1	30 days	30
Hydrochlorothiazide tbl. [28]	Hypertension	25	1	30 days	30

tbl. = tablets

cps. = capsules

In the essential medicines list (EML) of Cameroon, the health care facilities are divided into three main levels, i.e Health Centres, District Hospitals and Central Hospitals [23]. According to the EML, nine of the selected medicines should be available at all three levels of health facilities [23], while doxycycline, furosemide and hydrochlorothiazide are not meant to be stocked at the Health Centre level [23]. In the essential medicines list of the Democratic Republic of Congo the health care facilities are divided in two categories, Centre de Santé (CS) and Hopital General de Reference (HGR), and many of them are run or supplied by non-governmental organisations. The EML of the DR Congo [22] specifies that a HGR should stock all of the 12 medicines included in our study, while a CS should stock 9 of these medicines excluding atenolol, furosemide and hydrochlorothiazide. In practice, these rules are often not followed in either country.

### Selection of sampling sites

Medicine samples, and data on availability and prices, were collected from governmental health facilities, church health facilities, private pharmacies and informal (= illegal) vendors.

In the Democratic Republic of Congo, this study was conducted in the four provinces Ituri, North-Kivu, South-Kivu and Tanganyika, since the local partners involved in this study operated in these regions. From each of these four provinces, a complete list of health zones was obtained, comprising 37, 34, 34 and 11 health zones, respectively (total 116 health zones). Of these, 70 had to be excluded from this study since travel of the study personnel to and in these zones was unsafe according to the assessment of the local partners in the DR Congo. From the remaining 46 health zones, eight were randomly selected, two from each province, using the RAND-function of Microsoft Excel. The selected health zones were Nyankunde and Rethy (Ituri), Biena and Goma (North-Kivu), Nyangezi and Ruzizi (South-Kivu) and Kansimba and Nyemba (Tanganyika). Upon request by our local partners the Kadutu Health Zone in Bukavu, South-Kivu, was added to this selection since the central market of Bukavu is known as the biggest unlicensed market for medicines in this region, and the assessment of medicines quality there was of considerable interest.

In each of the selected health zones of the DR Congo, medicines were sampled from the Centre Hospital of that zone first. When the Centre Hospital was a governmental health facility, the geographically nearest church health facility, private pharmacy and informal vendor of medicines were identified and medicines were sampled also from these three sites. Correspondingly, if that Centre Hospital was a church health facility, the nearest governmental health facility, private pharmacy and informal vendor of medicines in that zone were identified and medicines were sampled from there. In the health zones Nynankunde und Rethy (Ituri Province) the central hospitals were under the authority of the government. However they are run by church-based organisations and obtain most of their medicines from faith-based drug supply organisations. Therefore in the data analysis they were classified as church health facilities. Furthermore in the same two health zones (Nyankunde and Rethy of Ituri province), no informal medicine vendors could be found, since medicine trade is under tight control in that province following a major international scandal involving falsified medicines [24]. Therefore, a total number of 34 medicine outlets, located in 9 health zones in four provinces were included into this study in the DR Congo. These included 10 Centres de Santé (CS) and 8 Hopitals General de Reference (HGR).

In Cameroon this study was conducted in 6 of the 10 regions of this country, i.e. Adamawa, Centre, Littoral, Northwest, Southwest and West, since the local partners involved in this study operated in these regions. A list of all church health facilities in these six regions was obtained, comprising 45 facilities. For each region, one church health facility was selected randomly, using the RAND-Function of Excel. Samples were collected in each region from this facility as well as from the geographically nearest governmental health facility, private pharmacy and informal vendor of medicines.

By chance, the random selection of six out of the 45 church health facilities included none of the ten facilities operated by catholic church organisations in these six regions. Upon request by the local partners, two out of the ten catholic health facilities were randomly chosen and additionally included in the Northwest and Centre regions. Therefore, a total number of 26 medicine outlets located in six regions were included into this study in Cameroon. According to our local partners, all selected medicines were expected to be available at each of the included health facilities.

### Collection of data and samples

Samples were collected in the Republic of Cameroon from August 2017 until November 2018 and in the DR Congo from July 2017 until May 2018. In public and church health facilities, the investigators identified themselves and explained the purpose of the study. In contrast in private pharmacies and illegal market vendors of medicines, samples were collected using a mystery shopper approach. For each medicine at each sampling site a quantity of up to 100 tablets or capsules was purchased if available (minimum 30 tablets or capsules). If several brands of the same medicine were available, the cheapest one was purchased. Samples were collected in their original packaging or containers if possible. At each of the 60 sampling sites, prices and quantities of purchased medicines were recorded on a standardized form. Sample number, brand name, batch number, manufacturing date, expiry date, name of manufacturer, international non-proprietary names (INN) of the active pharmaceutical ingredients (APIs), strength, dosage form, package size, and price were recorded as stated on the label, using a standardized form by the local staff.

### Calculation of availability, prices and affordability

WHO and Health Action International (HAI) have developed a standardized methodology for the investigation of medicines availability, prices and affordability [25]. The methodology was used in the analysis of the collected data. Availability was calculated as percentage of the sampling sites that had the requested medicine in stock in forms of tablets or capsules, irrespective of the strength [25]. Medicine prices were converted from local currency to US Dollar (USD) based on the exchange rates of January 2018 given by the European Commission [26]: Cameroon, 1000 West African CFA Franc (XOF) = 1.81933 USD; DR Congo, 1000 Congolese franc (CDF) = 0.63244 USD. As suggested by the WHO/HAI Manual [25], the observed individual prices were compared to an international reference price. For different types of medicines, the median of these price ratios was calculated, resulting in the median price ratio (MPR). The 2015 MSH median reference price (Supplier) was used for this purpose [27]. 502 medicine samples were collected in this study, but information on the individual prices of each sample was documented only for 476 of these samples. The remaining 26 collected samples could not be included into the price analysis, since e.g. only a summary price for several medicines bought at the same place had been recorded. For further 15 medicine samples, representing 500 mg amoxicillin / 62.5 mg clavulanic acid tablets, or 750 mg ciprofloxacin tablets, no reference price is given in the MSH price guide [27]. Therefore 461 medicine prices could be included into the analysis of median price ratios. Costs for a course of treatment were calculated using the treatment durations and dosage regimens shown in Table 1.

### Statistical analysis

Statistical calculations were performed using JMP version 14.2 (SAS GmbH, Heidelberg, Germany). To test for the statistical significance of median price ratios a Wilcoxon test was used for comparison of two groups.

### Ethical approval

This study was approved by the Ministry of Health of the DR Congo (Ref. CAB/Min-Prov/SGFEAHRAP/SK/01/2017), and by the Ministry of Public Health of the Republic of Cameroon, Comité National d’ Ethique de la Rechereche pour la Santé Humain (CNERSH), Ref. 243674339.

## Results

### Availability

**Fig 1:**
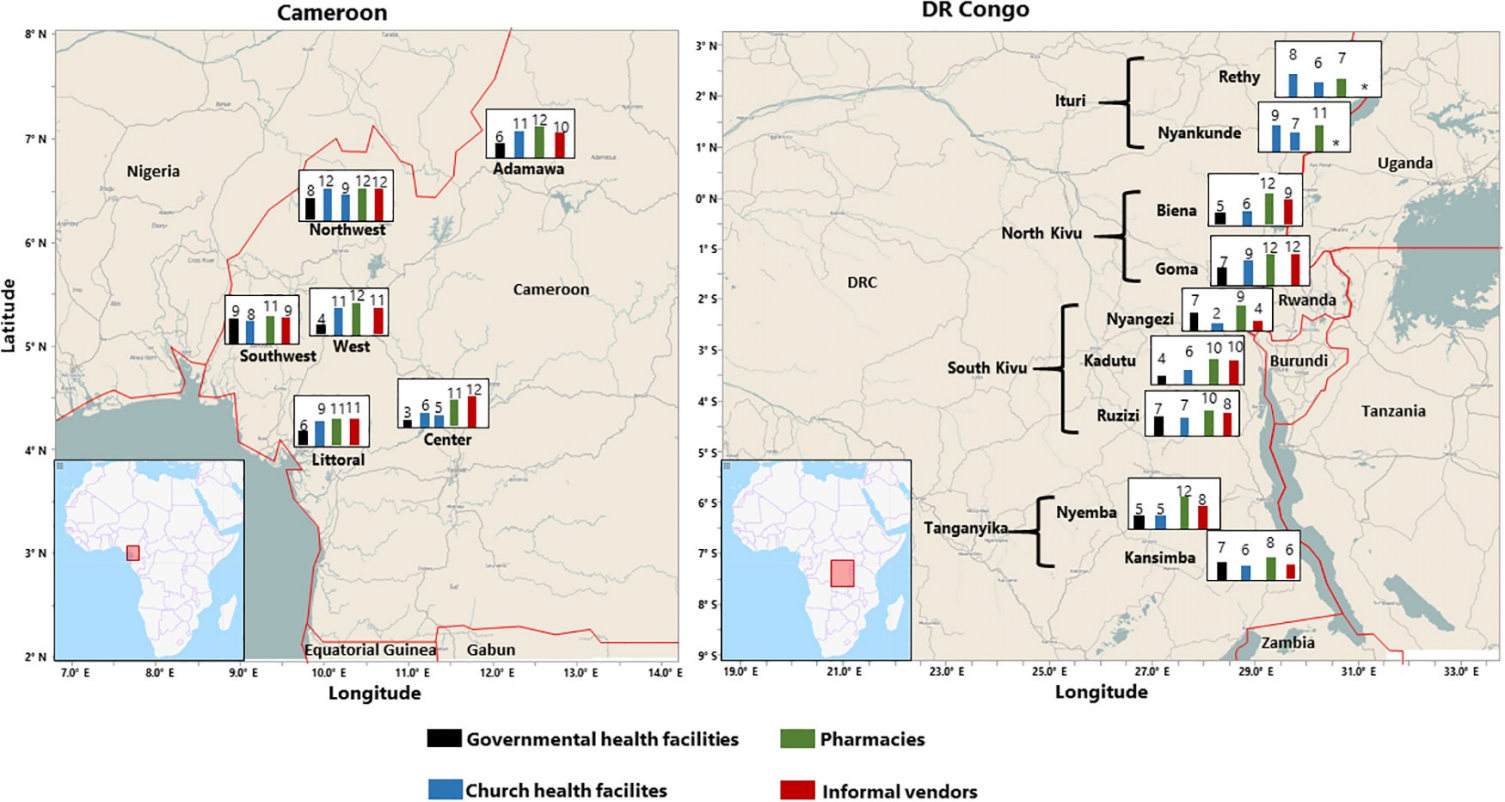
Map of the sampling regions, with numbers of medicines available at different types of facilities. 12 medicines were included into this study in each country. Therefore the maximum number of medicines recorded as available is 12. In the Ituri province in the DR Congo the health facilities under governmental authority were actually run by church organisations, therefore they were classified as church health facilities. * no informal vendor was identified in these health zones.

Fig 1 shows the approximate locations of the regions of Cameroon and the provinces and health zones of the DR Congo where data and samples for this study had been collected. A complete list of the medicines purchased at the sites, their characteristics and their prices is given in the S1 Table. Although we included only medicines which were comprised in the essential medicines list of these two countries, the full number of the 12 included medicines was only available in 6 of the 26 (23%) facilities in Cameroon and 4 of the 34 (12%) facilities in the DR Congo. The average availability of the 12 medicines was 78% in Cameroon and 64% in the DR Congo (Table 2). As obvious from Fig 1, no striking regional differences in medicine availability were observed within each country. Table 2 shows the detailed availability results of all investigated medicines in all types of facilities. Notably, government health facilities in the decidedly more affluent Cameroon are not better stocked than in the DR Congo (50% average availability in both countries). Therefore, the higher overall availability of medicines in Cameroon is attributable to the better stocks of church health facilities, pharmacies and informal vendors in this country. As expected, private pharmacy shops were better stocked than other types of facilities in both countries. Somewhat unexpectedly, informal vendors in Cameroon were extremely well stocked (93% availability), equal to licensed pharmacies.

**Table 2:** Medicine availability in the different types of facilities in Cameroon and the DR Congo.

	Cameroon										Democratic Republic of Congo									
INN	Governmental health facilities (n = 6)		Church health facilities (n = 8)		Pharmacies (n = 6)		Informal vendors		Cameroon, all (n = 26)		Governmental health facilities (n = 7)		Church health facilities (n = 11)		Pharmacies (n = 9)		Informal vendors (n = 7)		DR Congo, all (n = 34)	
											CS	HGR	CS	HGR						
							(n = 6)				(n = 4)	(n = 3)	(n = 6)	(n = 5)						
Amoxicillin / clavulanic acid	3/6		4/8		6/6		5/6		18/26	71%	0/4	1/3	1/6	0/5	6/9		4/7		12/34	37%
Amoxicillin	5/6		8/8		5/6		6/6		24/26	92%	4/4	2/3	5/6	5/5	9/9		7/7		32/34	94%
Ciprofloxacin	5/6		8/8		6/6		6/6		25/26	96%	4/4	3/3	5/6	5/5	9/9		6/7		32/34	94%
Doxycycline	4/6		6/8		6/6		6/6		22/26	85%	4/4	2/3	3/6	5/5	9/9		5/7		28/34	82%
Metronidazole	5/6		8/8		6/6		6/6		25/26	96%	4/4	3/3	6/6	5/5	7/9		7/7		32/34	94%
Penicillin V	0/6		2/8		5/6		6/6		14/26	56%	4/4	1/3	4/6	2/5	9/9		7/7		27/34	81%
Sulfamethoxazole / trimethoprim	4/6		7/8		6/6		6/6		23/26	89%	3/4	3/3	6/6	5/5	9/9		7/7		33/34	96%
Atenolol *											0/4	0/3	0/6	1/5	4/9		1/7		6/34	17%
Furosemide *	1/6		5/8		6/6		5/6		17/26	66%	2/4	1/3	1/6	4/5	9/9		6/7		23/34	69%
Glibenclamide	2/6		6/8		6/6		5/6		19/26	73%										
Hydrochlorothiazide *	1/6		6/8		6/6		6/6		19/26	73%	0/4	0/3	0/6	3/5	4/9		1/7		6/34	17%
Metformin	2/6		6/8		6/6		6/6		20/26	77%	0/4	0/3	0/6	3/5	7/9		2/7		12/34	33%
Salbutamol	4/6		5/8		2/6		4/6		15/26	57%	1/4	0/3	1/6	3/5	9/9		4/7		18/34	52%
											2.16/4	1.33/3	2.67/6	3.42/5						
Average	3.00/6		5.92/8		5.58/6		5.58/6		20.28/26		3.50/7		5.94/11		7.56/9		4.76/7		24.14/34	
	50%		74%		93%		93%		78%		50%		54%		84%		68%		64%	

*according to the EML, this medicine is not supplied to the CS-Level in the DR Congo [22]

**Fig 2:**
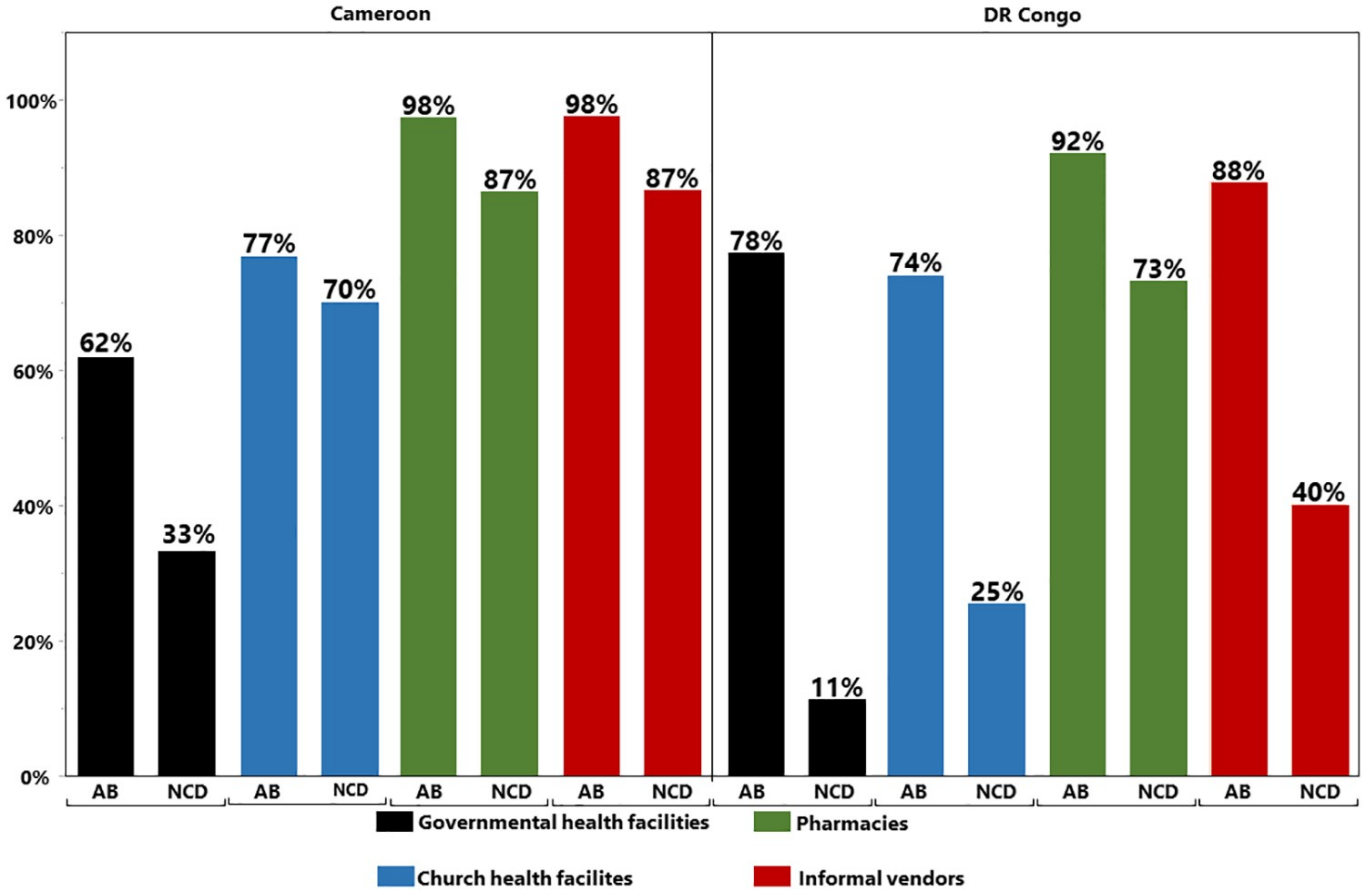
Availability of antibiotics and of medicines against non-communicable diseases in the four different types of health facilities.

Medicine availability was not very different in Centres de Santé (CS) and Hopitals General de Reference (HGR) (Table 2). Therefore we do not differentiate between these two categories in the following analyses. Fig 2 summarizes the availability of antibiotics and medicines against NCDs in health facilities of Cameroon and the DR Congo. Most striking is the poor availability of medicines against NCDs especially in government health facilities (33% and 11% availability in Cameroon and the DR Congo, respectively). In Cameroon, availability of these medicines in church health facilities reaches 70%.

### Prices

As explained in the Methods section, medicine prices recorded during the purchase of the samples were converted from national currency to USD, and medicines prices per unit (tablet or capsule) were calculated for each medicine. The ratio of the median price per unit to the international reference price given in the MSH International Medical Products Price Guide [25, 27] for the same medicine and strength was calculated resulting in the median price ratios (MPRs) shown in Table 3.

**Table 3:** Median price ratios for medicines and days wages’ needed for a course of treatment in Cameroon and in the DR Congo. The continent of origin of the medicines is given as stated on the label.

	Median Price Ratio (MPR)				Median Days wages needed for a course of treatment	
	Cameroon		DR Congo		Cameroon	DR Congo
	N	MPR	N	MPR		
**Amoxi/Clav**	12	3.20	6	4.57	4.34	10.05
**Amoxicillin**	23	3.03	30	1.61	0.87	1.00
**Ciprofloxacin**	21	4.88	29	1.70	1.00	0.84
**Doxycycline**	21	6.16	25	2.18	0.69	0.55
**Metronidazole**	24	5.37	29	3.11	0.62	0.78
**Penicillin V**	14	11.70	26	1.63	3.66	1.52
**Sulfa/Trimet**	22	3.50	30	1.77	0.54	0.56
**Atenolol**	0		6	10.66		2.68
**Furosemide**	16	13.42	23	3.89	1.12	0.67
**Glibenclamide**	18	13.57	0		2.11	
**Hydrochlorothiazide**	18	13.00	6	52.58	0.62	6.40
**Metformin**	19	4.25	12	7.35	2.60	8.53
**Salbutamol**	15	19.90	16	8.89	1.30	1.21
**Antibiotics**	137	4.47	175	1.80	0.87	0.88
**Medicines against NCDs**	86	8.81	63	7.11	1.24	1.61
**Governmental health facilities**	35	2.98	29	1.47	0.49	0.85
**Church health facilities**	62	6.10	65	1.78	1.16	0.88
**Pharmacies**	60	9.74	88	2.70	1.90	1.19
**Informal vendors**	66	4.25	56	2.22	0.61	0.84
**Africa**	18	6.84	39	2.37	0.64	0.94
**Americas**	2	8.52	0		0.92	
**Asia**	150	4.86	182	2.07	0.87	0.88
**Europe**	44	13.81	17	14.21	2.60	7.82
**not stated**	9	3.03	0		0.69	
**generic**	110	5.17	99	1.80	0.83	0.84
**branded**	97	4.88	129	2.38	0.99	1.00
**originator**	16	16.92	10	37.79	2.17	6.71
**All**	**223**	**5.69**	**238**	**2.17**	**0.99**	**0.94**

**Fig 3:**
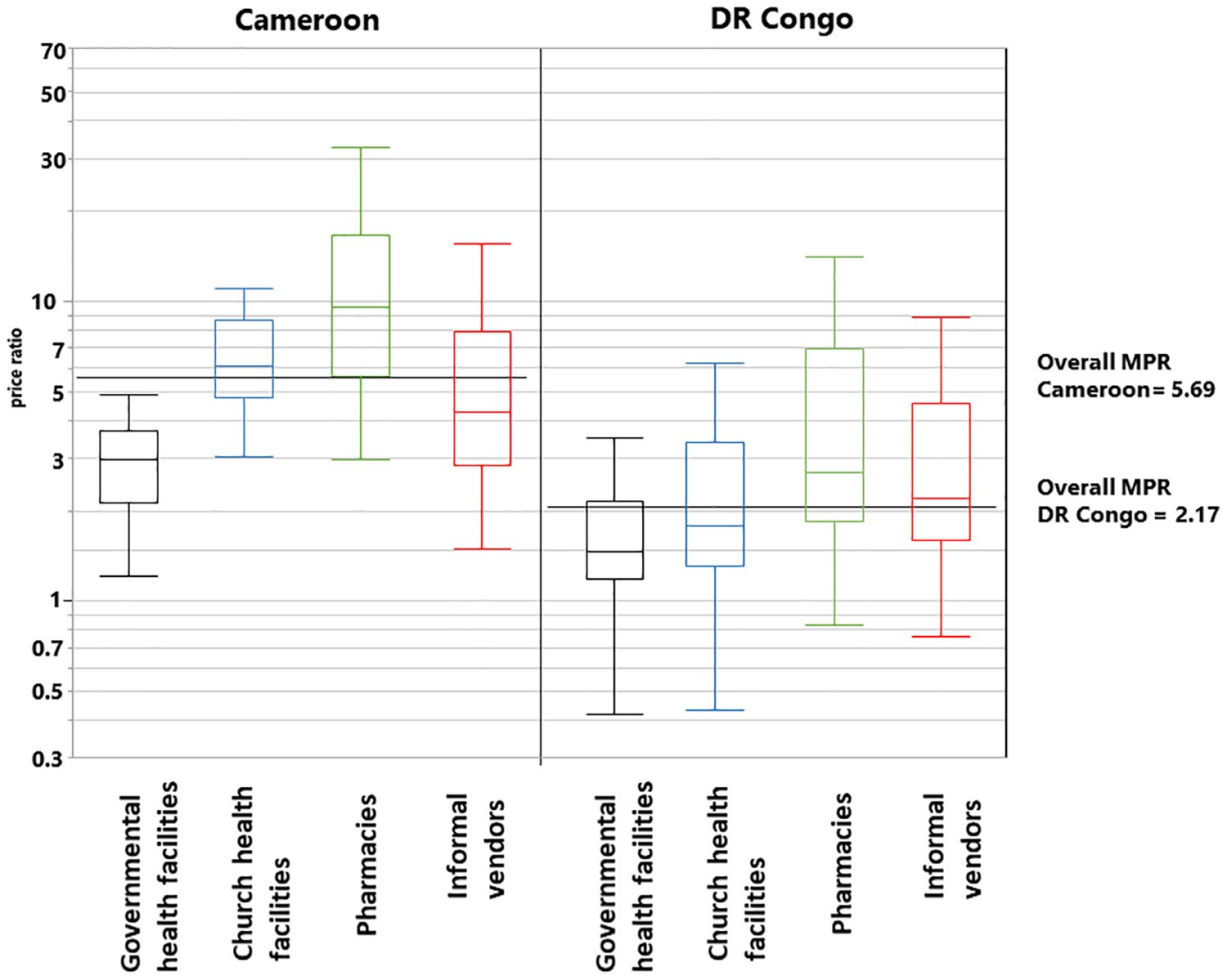
Boxplots of the price ratios in the four different types of facilities.

Overall, MPRs for Cameroon and the DR Congo resulted as 5.69 and 2.17 (p<0.0001), showing that medicines are more than twice as expensive in Cameroon than in the DR Congo. Table 3 and Fig 3 further show that medicine prices are higher in Cameroon in all four types of facilities, most pronouncedly in private pharmacy shops (MPR 9.74 in Cameroon vs. 2.70 in the DR Congo; p<0.0001). Fig 3 shows the range of the price ratios to the international reference price in the four different types of facilities. In the DR Congo, especially government health facilities offer some medicines at prices below the MSH reference price which is a price for international bulk procurement.

Fig 3 and Table 3 also show that in the DR Congo, government and church health facilities sell medicines cheaper than informal vendors. In contrast, in Cameroon medicine prices at church health facilities are higher than those at informal vendors (Fig 3).

**Fig 4:**
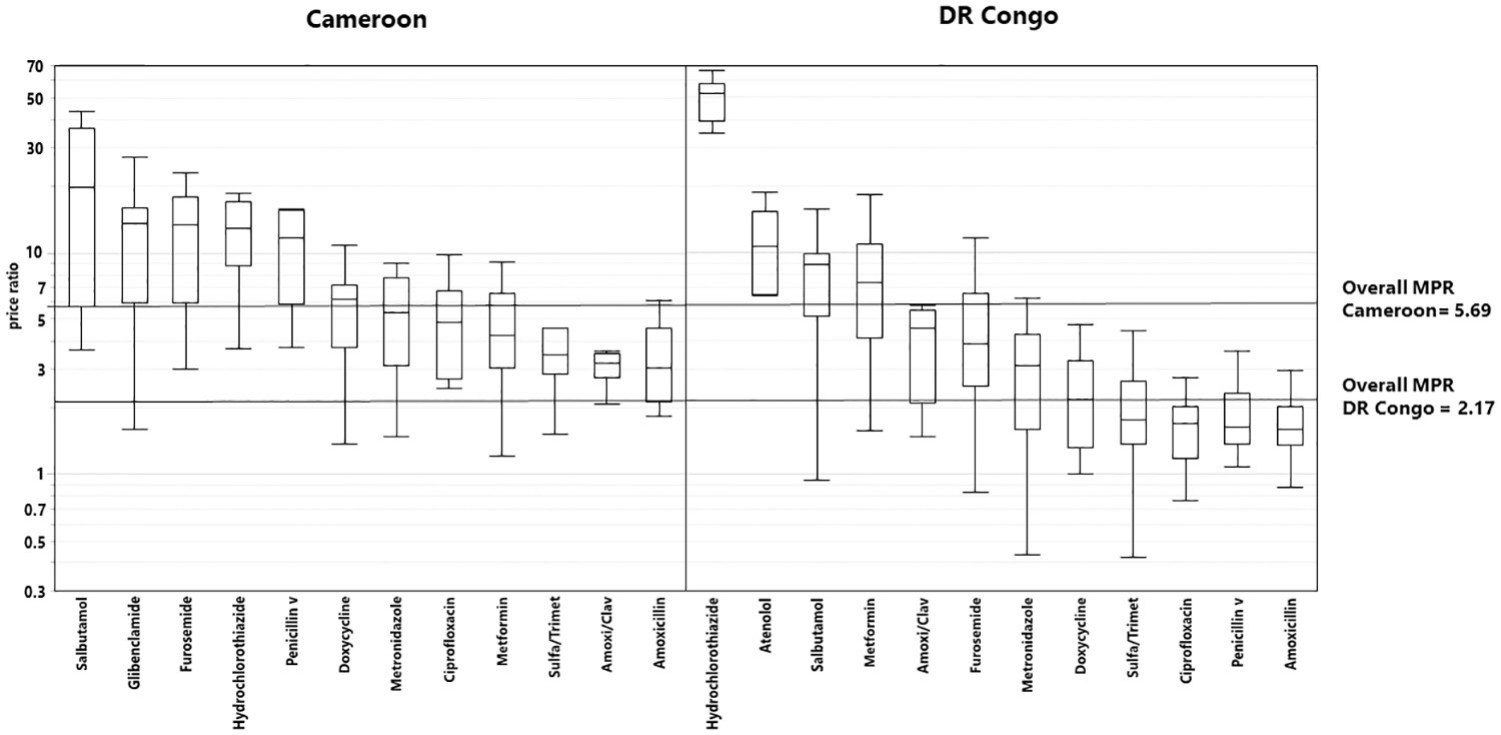
Boxplots of the price ratios of the collected medicines sorted by API and country.

Fig 4 shows the ranges of price ratios for different medicines in both countries. Medicines against NCDs show clearly higher MPRs than antibiotics.

Table 3 shows that antibiotics were clearly more expensive in Cameroon than in the DR Congo (MPR 4.47 versus 1.80; p<0.0001). In contrast, for medicines against NCDs, sometimes Cameroon was more expensive, sometimes the DR Congo, and altogether the MPR for medicines against NCDs was not significantly different in both countries (MPR 8.81 versus 7.11; p = 0.27).

In both countries, most medicines were generics (sold under their international non-proprietary names) or branded generics (off patent, but sold under a trade name given by the marketing authorization holder). Overall, only 6% of the collected samples represented originator medicines. As expected, these were very expensive, with MPRs of 16.92 in Cameroon and 37.79 in the DR Congo. The extremely high MPR observed in the DR Congo is strongly influenced by hydrochlorothiazide, which was solely available in the DR Congo in form of the originator medicine Esidrex^®^ at a very high price (MPR 52.58). As shown in Table 3, out of the 461 medicine samples included in the price analysis, 332 (72%) are manufactured in Asia and this proportion is similar in Cameroon and the DR Congo. However, the prices at which they are sold in both countries are remarkably different (MPR 4.86 versus 2.07, respectively; p<0.0001).

### Affordability

**Fig 5:**
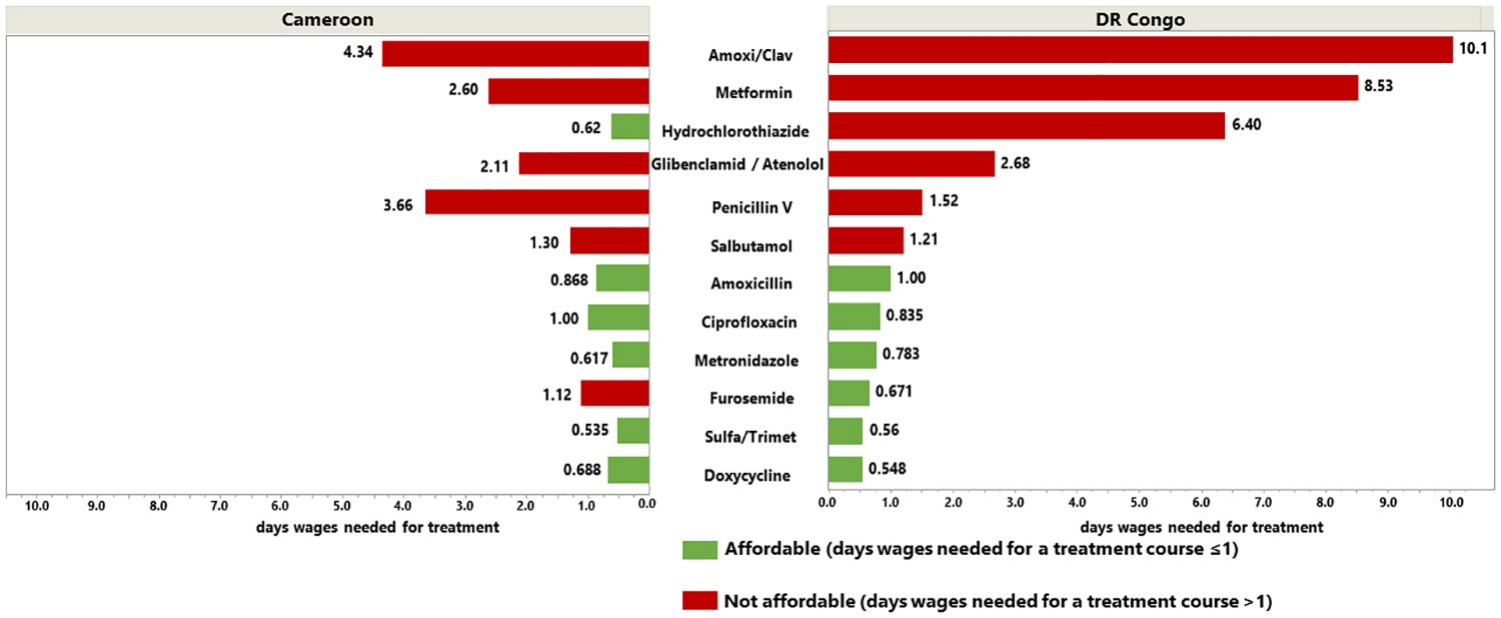
Median prices per treatment and median days’ wages needed for treatment.

As an indicator of the local affordability of a therapy regimen, the number of days´ wages needed to purchase a course of treatment was calculated following the WHO/HAI methodology [25]. The amount of tablets or capsules required for a course of treatment was calculated as shown in Table 1, Method section. For chronic diseases, the amount of medicine for 30 days of treatment was used [25]. Minimal wages set by the government of Cameroon and the DR Congo are given by the Country Reports on Human Rights Practices of 2018 [29]. The monthly minimal wage for Cameroon was divided by 30 [25], resulting in a daily minimum wage of 2.20 USD. For the DR Congo, we used the minimum daily wage of 1680 Congolese Francs (1.06 USD) which was in effect in January 2018. The Congolese Government has meanwhile started a stepwise increase of the minimum wage [29], but only after data collection for this study was completed. Table 3 shows the median number of days´ wages required for treatment with the different medicines investigated. According to WHO/HAI [25] a treatment course is considered unaffordable when it requires more than one day’s minimal wage. As graphically illustrated in Fig 5, several of the basic antibiotic treatment courses were affordable in both countries. In contrast, several of the treatments against NCDs were unaffordable, especially the important antidiabetic medicine metformin.

As shown in Table 3, medicines sold in pharmacies were less affordable than medicines sold in the other types of facilities. While treatments with medicines produced in Africa and Asia were affordable in many cases, those with medicines imported from Europe were not. Likewise, treatment courses with generic medicines were affordable, while treatment courses with originator medicines were not.

## Discussion

With price data from a total of 461 medicines purchased at 60 sampling sites this study provides valuable new insight into the availability and affordability of essential medicines in Cameroon and the DR Congo.

Prior to this study, only a single comprehensive investigation of medicine availability in the DR Congo had been carried out, as part of the “Service Availability and Readiness Assessment” (SARA) published by the DR Congo Ministry of Public Health in 2014 [16]. This SARA included data on 20 medicines, most of them against NCDs. In contrast to our study, it did not include private pharmacies and informal vendors. For government and church (or private) health facilities, it reported an average medicine availability of 20%, which is in agreement with our findings on the availability of medicines against NCDs in these kind of facilities (Fig 2). The SARA included only four antibiotics, three of them as injectable solutions and one as oral suspension. In contrast, our present study included six first-line (“ACCESS GROUP”) and one second-line (“WATCH GROUP”) antibiotics in form of capsules or tablets [30]. It is important and encouraging that, with the exception of the expensive amoxicillin/clavulanic acid, these antibiotics showed good availability in the health facilities of the DR Congo. This is in agreement with the finding by O’Connel et al. [15] that first-line antimalarials showed good availability in public health facilities of the DR Congo. In contrast to the SARA [16], our study included pharmacies and informal vendors, showing that they accomplished a higher medicines availability than government and church health facilities. This should be seen as a motivation to further improve availability in government and church facilities. To the best of our knowledge, no comprehensive analysis of medicine prices and affordability has been published from the DR Congo so far. In other low- and middle-income countries, median price ratios have been reported to be, on average 3.1 in the public sector and 5.3 in the private sector [31]. In comparison, median price ratios are remarkably low in the DR Congo (Table 3), indicating either successful cost-effective procurement, or subsidies to medicine prices, or both. Especially government and church health facilities in the DR Congo sell medicines cheaper than informal vendors. This is desirable from a public health perspective, in order to minimize the incentive for patients for to buy from informal vendors where medicine quality problems are expected to occur more frequently.

In Cameroon, prior to our present study one previous comprehensive investigation of medicine availability and prices has been published [14]. It investigated 22 medicines against cardiovascular diseases and diabetes, four of these (furosemide, glibenclamide, hydrochlorothiazide and metformin) were also included in the present study. The overall availability of each of these four medicines reported by Jingi et al. [14] is similar to the one reported in our study for Cameroon. However, our study showed lower availability in government health facilities. This may indicate a deterioration of medicine availability in the government facilities of Cameroon since 2012. However, informal vendors in Cameroon stocked and readily sold all investigated antibiotics including the expensive amoxicillin / clavulanic acid tablets. In view of the increasing danger of increasing antimicrobial resistance, this is a worrying finding.

Just as we did in our study, Jingi et al. [14] reported median price ratios of the medicines investigated relative to the international reference price published by MSH [27]. The overall MPR from the data published by these authors results as 5.97, similar to the overall value of 5.69 which we show for Cameroon in Table 2. For the individual medicines, the MPRs reported by Jingi et al. [14] range from 0.59 to 70.81. Notably, for the four medicines furosemide, glibenclamide, hydrochlorothiazide and metformin the authors report lower MPRs and consequently better affordability than reported in our present study. Closer investigation showed that the MPRs for these four medicines which we recorded at government health facilities were, in fact, quite similar to those reported by Jingi et al. who mainly focused on government facilities. However, since only few government facilities had these medicines available, in our study most (92%) of the prices of these four medicines derive from pharmacies, church health facilities and informal vendors, where medicines are substantially more expensive than in government facilities. The overall higher MPRs of medicines from private pharmacies may reflect the presence of a more affluent middle class in Cameroon which is able to afford more expensive medicines in private pharmacy shops.

As specific reason for concern resulting from our study is the overall low availability and affordability of medicines against non-communicable diseases. The WHO Global Status Report on NCDs 2014 [32] emphasized that the burden of disease regarding NCDs is heavily concentrated in low and middle-income countries. Hunter-Adams et al. [33] expect that the burden of diabetes in Africa will increase till 2035 by 110%. Also a study from Tsabang et al. [34] revealed that the prevalence of diabetes and hypertension is increasing in Cameroon. Since medicines against NCDs showed only 11% availability in governmental health facilities in the DR Congo and 33% in the governmental health facilities in Cameroon, drastic improvements are needed to ensure that medications against NCDs are available and affordable for the population, and that health care staff is trained in diagnosis, management and the treatment of NCDs. Notably, medicines against NCDs were well available in the private pharmacies in Cameroon and in the DR Congo and in informal vendors in Cameroon.

The affordability of most of the available medicines against NCDs was low. For a 30 days’ treatment with metformin, 4.9 days wages in DR Congo and 2.8 days wages in Cameroon would be necessary. In both countries a major share of health care expenditures has to be paid by patients out of pocket [35, 36]. Notably, a 2019 survey indicated that high prices of medicines were a prominent cause for non-adherence among diabetes patients in two hospitals in Cameroon [37]. This underlines the importance of the affordability of essential medicines as a core priority in achieving universal health coverage, as already highlighted by Wirtz et al. [38].

In our study, the most expensive medicines were originator medicines, produced mainly in Europe. Hydrochlorothiazide in the Democratic Republic of Congo was solely available as Esidrex^®^ (Novartis) in private pharmacies at an extraordinary high price 53 times higher than the MSH reference price. This may drive the population to buy hydrochlorothiazide from illicit sources, providing medicines of doubtful quality.

## Limitations of this study

This study followed the WHO/HAI methodology [25] in data analysis but not in data collection, since it was part of a medicine quality study. We collected the price of the cheapest brand for each of the 12 requested medicines in each of the sampling sites, and did not ask for both the lowest priced generics and the lowest price originator for every medicine in every sample site, as foreseen by the WHO/HAI methodology [25]. Also most of our sampling sites were located in small or large towns, therefore this survey might not reflect the situation for the rural population in Cameroon and the DR Congo correctly. Since the medicines selected were antibiotics, cardiovascular medicines and antidiabetics, the results are not representative for other therapeutic classes such as antiretrovirals or cytostatics. This survey measured medicine availability and prices only at one time point and does not reflect changes over time. Data analysis has been limited to descriptive statistical methods. The authors are carrying out additional studies on availability, prices and quality of medicines in Africa, and intend to present additional data analyses once these studies have been completed.

## Conclusion

The average medicine availability in Cameroon and the Democratic Republic of Congo is lower than the 80% quota targeted by the WHO. Despite the high availability of antibiotics in the private sector, the availability of medicines in the public sector requires improvement in both countries. Also regarding the affordability, further improvements are necessary at least for some of the investigated medicines. This is especially true for the medicines against non-communicable diseases, in order to ensure availability and affordability.

## Supporting information

S1 Table (XLSX)
